# How do general practice residents use social networking sites in asynchronous distance learning?

**DOI:** 10.1186/s12909-015-0435-x

**Published:** 2015-09-21

**Authors:** Hubert Maisonneuve, Juliette Chambe, Mathieu Lorenzo, Thierry Pelaccia

**Affiliations:** 1 Primary Care Unit, Faculty of Medicine, Geneva, Switzerland; 2 Department of General Medicine, Faculty of Medicine, Strasbourg, France; 3 Health Sciences Education Research Laboratory, Department of Medical Education, Faculty of Medicine, Strasbourg, France

## Abstract

**Background:**

Blended learning environments - involving both face-to-face and remote interactions - make it easier to adapt learning programs to constraints such as residents’ location and low teacher-student ratio. Social networking sites (SNS) such as Facebook®, while not originally intended to be used as learning environments, may be adapted for the distance-learning part of training programs. The purpose of our study was to explore the use of SNS for asynchronous distance learning in a blended learning environment as well as its influence on learners’ face-to-face interactions.

**Methods:**

We conducted a qualitative study and carried out semi-structured interviews. We performed purposeful sampling for maximal variation to include eight general practice residents in 2^nd^ and 3^rd^ year training. A thematic analysis was performed.

**Results:**

The social integration of SNS facilitates the engagement of users in their learning tasks. This may also stimulate students’ interactions and group cohesion when members meet up in person.

**Conclusions:**

Most of the general practice residents who work in the blended learning environment we studied had a positive appraisal on their use of SNS. In particular, we report a positive impact on their engagement in learning and their participation in discussions during face-to-face instruction. Further studies are needed in order to evaluate the effectiveness of SNS in blended learning environments and the appropriation of SNS by teachers.

**Electronic supplementary material:**

The online version of this article (doi:10.1186/s12909-015-0435-x) contains supplementary material, which is available to authorized users.

## Background

Post-graduate training allows future general practitioners to develop the skills needed to provide efficient care [1, 2]. Teaching general practice meets new challenges and issues in several countries, including France [3]. In particular, in France, general practice departments face a growing number of students to train with a current ratio that ranges from 46 to 210 residents for 1 teacher [4]. Furthermore, general practice residents are often assigned for practice to rural community hospitals or district general hospitals and thereby dispersed over wide geographic areas [5, 6]. Distance learning has the potential to address the constraints of a low teacher-student ratio and the geographic dispersion of residents [7].

Synchronous distance learning occurs when teachers and students interact simultaneously in different places. Asynchronous distance learning occurs when teachers and students interact in different places, at different times. Asynchronous distance learning is more adapted to medical students with oftentime incompatible schedules. Nevertheless, distance-learning environments are ill adapted for developing certain skills, such as clinical and interpersonal abilities. It is therefore possible to design blended learning environments. According to Garrison and Kanuka, blended learning refers to the systematic integration of online and face-to-face engagement to support and enhance meaningful interactions between students, teachers and resources [8]. Blended learning enables residents to develop reflective thinking skills, clinical skills, clinical reasoning and self efficacy [9].

Electronic platforms, also known as learning management system (LMS) or virtual learning environments, are widely used for distance learning [10]. In the field of education, Moodle is the most widely used LMS [11]. Thanks to LMS, teachers can provide scientific content to learners, and enable supervision of both the group as a whole and the individual learner. Furthermore, LMS facilitates long-distance collaboration between learners [8]. On the other hand, LMS are difficult to implement, since they require information technology team support for installation, updates, backups, maintenance and technical assistance [12]. The amount of time needed by teachers and students to master the system may reduce the time spent on actual learning activities. Moreover, previous studies have shown that sometimes, the design of e-learning platforms reduces the efficacy of these platforms for teaching [13].

Social networking sites (SNS) such as Facebook® may be used as learning tools to address the limitations of traditional electronic platforms for several reasons [14]. First, data can be accessed using multiple devices (such as computers, smartphones and tablets), which allows users to work more flexibly. Second, as the host manages the technical administration, teachers don’t need to focus on this issue. Finally, students may master SNS easily as in many cases they already use it in their private lives [15, 16].

The use of SNS for teaching and learning has already been studied. The objectives of previous researches were mainly to discuss about privacy and professionalism [17-19], to evaluate the users’ satisfaction or SNS benefits to enhance empathy, reflection, clinical skills or knowledge [20-22]. However, the way medical students use SNS for learning and their experience as users have been poorly studied, especially in postgraduate general medicine training. We therefore chose to explore how residents use SNS to train for their future career.

To achieve these goals, we drew on the concept of social appropriation. This concept is relevant to the field of social networking since it explores both the use of a digital tool and its integration into learners’ daily lives. Social appropriation needs to meet 3 conditions. First, there must be “technical and cognitive command of the artefact” [23], which requires a “minimum level of technical competence”[23] regarding the technical object. Second, a “meaningful integration of the technical object in the user’s everyday practice”[23]. And third, a “repeated use of the technology, which opens the way to creativity” [23].

The aim of the present study on general practice residents’ social appropriation of SNS such as Facebook® was to understand their use of SNS in learning, as well as its influence on interactions between learners during face-to-face learning sessions.

## Methods

### Background

The goal of our study was to explore, analyse and understand the users’ experience of SNS in a learning context. We therefore chose a qualitative approach, aiming to explore the behaviours, feelings and experiences [24] of SNS users in these kinds of situations. We carried out semi-structured interviews with general practice residents at the Faculty of Medicine of Strasbourg. We recruited participants on a voluntary basis and asked them to sign a consent form containing information on the study objectives and procedures, the voluntary nature of participation, foreseeable risks, expected benefits and data confidentiality methods. The Strasbourg University ethical review committee approved the study protocol.

### Study design

In France, general practice is a postgraduate program. After graduating from medical school, residents attend a 3-year general practice-training program in one of the 33 general practice departments across France. This program consists of 2 to 2,5 years in hospital departments and 0,5 to 1 year supervised general practice. Residents are located all around different regions and rotate each semester. During this period, they also attend theoretical courses consisting of 200 h.

The context of our study was a blended learning environment created in 2011 at the general practice department of Strasbourg University. The blended learning environment we studied was built to meet several educational objectives: professional identity development, clinical reasoning, decision-making, therapeutic education, pluridisciplinarity and ageing, private practice requirements, etc. It consisted of 6 sessions, 1 during each semester. We tried to create an interactive community of practice [25] between groups of 15 to 20 residents supervised by 1 teacher in order to share medical knowledge and best practices [26] through the presentation of personal projects during face-to-face meetings. The face-to-face part of the program included 12 h of training per semester (4*3 h). The subject and the way the project was going to be presented were decided at the beginning of the program. Each project could take varied forms: clinical situation reports, case studies, literature reviews, building role-play scenarios, etc. An important aspect was to increase interactions between participants through a creative presentation of the projects. The main purpose of the distance part was to prepare each participant’s project as well as the face-to-face sessions. Among the 13 teachers of the general practice department, 2 teachers (HM and FR), both medical doctors holding master degrees in medical education created a virtual community of practice using (Facebook®) for asynchronous distance learning. A closed (Facebook®) group was created to this end. Residents were asked to post the building blocks they had used to create their projects, their references, to read others residents’ posts and provide feedback when possible. Residents were free to use e-mail instead of SNS to communicate with the supervisor and receive feedback. Participation in the virtual community of practice was not compulsory and the participation in the virtual community didn’t influence residents’ evaluations. We asked students to make all patient-related data they published anonymous. The supervisors checked for anonymity each week.

### Study population

During the study period, four hundred residents were studying general practice in the Strasbourg region. We included residents who had attended at least two training sessions: one using SNS for asynchronous distance learning and the other using e-mail. Our target population was 40 residents. We used the principle of maximal variation of the use of (Facebook®) for personal and teaching purposes. We decided to proceed in this way because our study was exploratory. To reach maximal variation, we checked (Facebook®) profiles of potential participants. For example, we tried to target digital native users [27] constantly active on SNS as well as light users whose (Facebook®) profiles were nearly empty. We contacted participants using SNS, phone or e-mail, 7 to 14 days after the end of the teaching session. This delay was intended to give participants some distance and perspective on their experiences in the learning environment. We sent to participants an explanatory note about the study by email The volunteers chose the interview location. The interview time was agreed upon by the volunteers and HM.

We stopped recruitment when we reached data saturation, which means that no new elements emerged during the data analysis phase [28, 29].

### Interviews

HM carried out all the semi-structured interviews based on an interview guide. The interview guide (Additional file 1) was written by HM, based on the concept of social appropriation. It aimed to document, in particular, the experience of using SNS, mastering it, ways of using it, how each interviewee contributed to carrying out his or her personal project, interactions with peers, and feelings when faced with the tool and its integration into the learning environment. The user’s age was also recorded, as well as the number of learning sessions attended by residents using SNS. At the end of the interview, the interviewees were invited to comment freely on using SNS in a learning situation.

The interview guide was tested during a preliminary interview with residents. We modified it gradually as data were analysed, as recommended in the practice of qualitative research [29]. The following are some examples of questions HM asked during the interviews:

- “How do you use (Facebook®) for your work?”
- “Do you remember specific moments when you were working on it?”
- “Where could you access (Facebook®)?”

Each interview was recorded in audio format.

### Data analysis

HM listened and fully transcribed the interviews. Names and personal information were changed. HM and ML (medical doctor, holding a master degree in medical education) then performed a three-step thematic analysis, using NVivo 10® with the purpose of coding, and developing categories and themes from the participants’ responses: 1) we carried out a parallel-blinded coding process on all the verbatim accounts 2). The codes were then grouped into categories. Associations were sought between these categories in order to specify their connections. Identifying these categories enabled certain themes to emerge, which we mapped out 3). The themes, categories and connections established were compared with data from previous publications. HM and ML independently reviewed the coding several times and categorized data to ensure exhaustion of codes. They then compare results, refined the coding and categorized data, and identified major themes. In the case of disagreement, they reached a consensus through discussion from within the set of applicable themes that had been developed. We aimed to reach a 90 % inter-coder accuracy rate (rate between the agreement number and the sum of agreement number and disagreement number), as recommended by Miles and Huberman [29].

## Results

**Fig. 1:**
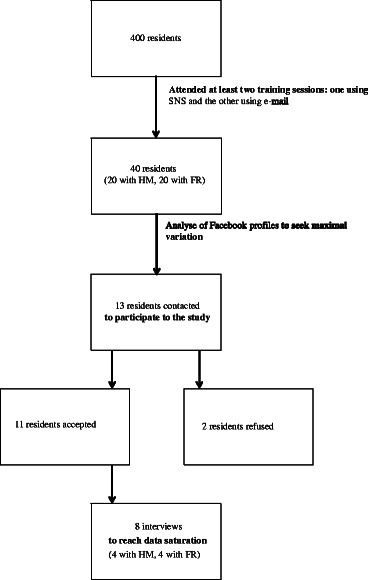
Flowchart of the study population

Thirteen residents were contacted. Eleven agreed to participate in the study. Eight interviews were carried out to reach saturation (Fig. 1: Flowchart of the study population). The participants’ age ranged from 24 to 30. Five were females and three were males. They had attended one to three learning sessions using SNS. They were in 2nd or 3rd year of residency.

We identified five main themes and used them to present our results:

- The border between professional and personal spheres of SNS use was blurred;
- Checking SNS and posting on SNS were two distinct activities;
- Using SNS stimulated and facilitated work on users’ personal projects;
- Exchanges on SNS were limited, but residents posted with the intention that their posts would be read;
- The use of SNS between face-to-face learning sessions fostered group cohesion and face-to-face exchanges.

### The border between professional and personal spheres of SNS use was blurred

All the residents we interviewed used SNS in their private lives. Furthermore, most of them had already used it in a prior learning context.

The border between private and professional spheres was blurred. Private elements could easily intrude into the professional sphere:

- “*Some of my friends are doctors, and they sometimes post things that I can share with others who aren’t in the medical field*.” (Resident 2)
- “*When I was in x [city name anonymized] […], I was in charge of communication […]. I used this position to spread the word about events and other activities related to the residency*.” (Resident 6)
- “*In my personal life, with my friends and family, I usually prefer phoning [to communicate]. In my professional life, I’d rather use e-mail and Facebook*® *[…]. It’s actually a kind of mix.*” (Resident 5)

Conversely, the professional sphere could also intrude into the private sphere. residents didn’t complain about this:

- Interviewer: “*So for you, professional elements may enter into the personal sphere, but in the end, as long as it’s a choice…”*
- Resident 7: “*It doesn’t bother me.”*

The interviewees explained this by the fact that they could choose whether or not to respond to requests on SNS.

According to Resident 4, working with SNS was impossible because of the “entertaining” connotation associated with the tool. He explained this impediment as resulting from his personality and his need for structure with a clear delimitation between personal and professional activities: “*I cannot associate Facebook® with the professional sphere […] This is for entertainment*” (Resident 4)

### Checking SNS and posting on SNS were two distinct activities

When using SNS for private purposes, residents checked (or read) SNS more often than they posted on it. Usage was thus more often passive than active. This was also true when using SNS for professional purposes.

Users checked SNS from different locations: in public transport, on a café terrace, in a doctor’s waiting room, in bed while one’s partner was sleeping, or on a public computer at an internet café. Users were most likely to check SNS during breaks in professional activities, when they were bored, or when they received notifications that other group members had posted something. The participants said that they checked SNS on their computer or phone. They preferred the phone for its mobility and the computer for its readability: “*When I just want to see what’s going on […], I can do it from anywhere on my phone*” (Resident 6).

Checking SNS was easy, spontaneous “*It’s a spontaneous use*” (Resident 6), and regular. On a Smartphone, users checked SNS much as often as text messages or e-mails. “*I check when I want, or when I’ve nothing special to do; Actually, it mainly happens when I’m bored […] Facebook is a mix between MMS, SMS and the telephone*” (Resident 5)

Posting mainly took place when a user was working on his or her personal project. This activity functioned differently than checking. A specific time was dedicated to posting, and the preferred place was home, at a regular workstation, on the resident’s personal computer:

- “*Working […] always happened at home because that way I could take my time and look at what I wanted to look at.*” (Resident 6)
- “*[Working] is something I do on a computer, not on the iPhone, and that means being at home. Usually it’s at night: the baby’s asleep and my girlfriend's watching television, so that's the time I have for myself.*” (Resident 5)

Some residents stressed the flexibility the tool gave them in terms of timing:

“*We can [work] whenever we want to - we can organize our work however we want. If I want to post something at midnight because I’m motivated or I have an idea, you know, I post it at midnight.*” (Resident 3)

### Using SNS stimulated and facilitated work on users’ personal projects

Nearly all participants explained how the collaborative use of the tool - eased by spontaneous exchanges on SNS and their freedom to use it where, when and as they pleased - helped them with their personal projects. Using SNS seemed “*more natural than e-mail*”, which let them “*make progress [on work]*” (Resident 1).

Participants felt that they spent more time on their personal projects in the classes where they used SNS: “*I think that when we use Facebook*® *as a learning tool, we spend a lot more time connected to our subjects than in a normal learning environment.*”

They explained this by the fact that you can work “*as you go along […] because you spend 5 min here and 10 min there, so it doesn’t feel like I’m spending time on it*” (Resident 3).

Furthermore, a healthy spirit of competition was generated when residents read what others had produced on SNS, as Resident 3 emphasized: “*The fact that others post […] as they work [leads to] emulation, because we see that other residents are thinking, reflecting on and building something.*“ Sharing resources therefore seemed enriching. The speed of interactions between residents or between residents and teachers was also found to be helpful, and the teacher’s presence on SNS encouraged participants to work. “*[The teacher] is very active, he manages the stuff, he points out the things to discuss during the face-to-face sessions […]. He helps us when we lack knowledge and advises us on which directions to take*” (Resident 7)

According to Resident 7, since one of the goals of the personal project was to stimulate interactions within the group, the teachers wanted residents to be “*creative about the presentation of their work*”, meaning that they should find “*an original way to present [their] work*”. According to the interviewee, residents were not familiar with this type of request. They sometimes found it “*stressful”.* She agreed that “*[she] needed to be creative to be interesting to others, and [she] knew that it is part of adult teaching, because we can’t teach an adult like a child”*. But she also considered that “*not everyone has the requisite resources to be creative*” She believed that the difficulties they met were *“due to the kind of speech* [students] *were used to hearing in their pre-graduate studies: rigorous, strict, objective. But once in postgrad, you needed to be creative*”. According to the interviewee, SNS was thus an interesting tool to encourage creativity, since the nature of the tool itself enabled participants to interact with each other easily. Using SNS enabled them to overcome an impediment to investing themselves in their personal projects.

### Exchanges on SNS were limited, but residents posted with the intention that their posts would be read

The interactivity between the tool and the student, described as an asset for the personal project, rarely gave way to online discussions:

“*Not many people comment. They only comment during meetings - I mean, when we’re all physically together in class. In those situations, we had exchanges, but on FB, not really.*” (Resident 5)

Despite the limited nature of these interactions, some residents wrote with the objective of being read and understood by others. They posted summaries of their research as well as their sources:

“*When you post short things, people read them. That’s why I made PowerPoints*®*, too, because otherwise the content would have been too broad […]. It has to be easy to take in.”* (Resident 5)

### The use of SNS between face-to-face learning sessions fostered group cohesion and face-to-face exchanges

SNS also allowed users to “*keep in touch*” (Resident 1) with the group between each face-to-face session. This created a form of cohesion within the group:

“*We saw each other once, then one more time, and in between there was nothing at all. So it helped us to stay in touch. Facebook helped to create cohesion within the group, that’s for sure*” (Resident 1).

Seeing the work of their peers allowed residents to adapt the content of their own projects in order to avoid redundancy. “*When we saw that someone had already discussed this point in his work, we adapted our speech to be concise in relation to this point. Sometimes, we were thinking: he discussed it this way, I’ll then search this information and discuss it from a different perspective*.” (Resident 1).

Knowing what other residents were working on helped residents to adapt the content of their projects to avoid redundancy as well as to have in-depth discussions during face-to-face sessions:

- “*Normally, everyone read what was discussed on Facebook*®*. I personally find that the discussions we have after previous discussions on FB are much richer and more detailed than the discussions that come up when we just present our little PowerPoint*® *at the end of class*.” (Resident 7)
- “*I really liked the classes with social networks because I felt like that made the class more lively and that we were more motivated to participate in it.*” (Resident 5)

## Discussion

Our study suggests that the level of social appropriation of SNS by the residents we interviewed can be explained by the fact that the tool is already a part of their daily lives. The use of SNS also generated a healthy spirit of competition within the group. SNS was a natural way to present creative work without having specific creative skills. Perceived working time was longer in this model than in traditional learning environments, without being a cause for complaint among residents, since the SNS gave them the opportunity to work only when they wanted to. Residents checked their peers’ work but rarely commented on it. The personal work carried out over SNS had a positive impact on face-to-face learning sessions since it fostered exchanges when residents met up.

### A high level of social appropriation

According to the concept of social appropriation, users must demonstrate a minimum level of technical competence regarding the tool in order to post their personal work and to consult that of others. This technical competence must also be creatively and significantly integrated into their daily practices [23].

In our study, the 3 conditions we described in the background were met: 1). It seemed that the use of SNS was not associated with any technical difficulties 2). Technical mastery of the tool enabled learners to integrate it into their daily lives 3). As part of their approach to completing a personal project, the rules for creativity were respected. A previous study with general practice residents showed that the perceived usefulness, ease of use and intention to use SNS for work or training were scored positively [30]. The blurred boundary between social and educational use, which is needed to meet the 2^nd^ and 3^rd^ conditions of social appropriation, were also already observed in a population of medical and pharmacy students [31, 32].

Our study focused on students’ point of view. Further research might explore teachers’ appropriation. To reach this goal, the framework described to help teachers appropriate SNS for teaching purposes [33] may be useful.

Most Facebook properties and functions used in this study also exist in other LMS such as Moodle. Using these platforms would lead to a loss of the benefits of Facebook in terms of user familiarity but might gain much more in functionality and active engagement. Exploring social appropriation of Moodle users with a similar method could be another research objective in this field.

### An infrequent instrumental conflict

In a given context, when a human being interacts with an object for the first time, he or she spontaneously attributes a function to it. However, the function attributed to the object is not necessarily the function for which it was designed. Instrumental conflict is referred to when the learner does not attribute an educational function to a digital learning object [34]. Students using SNS in a learning context must therefore attribute an educational function to the tool. In our study, this was the case for nearly all the residents interviewed. It was easy for most of them to attribute educational functions to SNS, in particular those of communication, interaction and collaborative work with their peers.

In our study, one student refused to use SNS for this purpose. Similar students have been identified in a previous study that explored SNS usage by pharmacy students [31]. The prevalence of these students who are reluctant to use SNS for teaching requires further evaluation.

### A way to decrease students’ stress when asked to present their work in an original way

SNS was considered as a useful way to present creative work without having creative skills. With an increase in the students being ‘stressed’ when asked to find an original way to present their work, SNS may be an interesting tool to give students this opportunity while at the same time reducing their stress levels when performing such a task.

### Two distinct levels of cognitive engagement during asynchronous distance learning work

Cognitive engagement is defined by the degree of attention, concentration and mobilization of mental resources with the aim of acquiring new knowledge and skills. It is reflected in the deployment of learning strategies [35].

Based on our findings, we can identify two situations that correspond to two distinct levels of cognitive engagement. One involves “checking” on SNS, and the other relates to “posting” on SNS. Checking was performed in a more passive way and required limited cognitive engagement. Residents reported that they could check from anywhere and at any time, as the opportunity arises. Posting required a more significant cognitive engagement, with residents dedicating a specific time to this activity, in a well-defined context.

In a face-to-face problem-based learning environment, engagement increased primarily in two situations: while completing a personal project and during group discussions [36]. In the learning environment we studied, the activity of completing the personal project may have led residents to gain knowledge related to their subject, by consulting both resources provided by the teacher and work done by other residents. The cohesion and group dynamics seemed to be improved by the use of SNS. These circumstances are recognized as fostering cognitive engagement in learning [36]. They may explain why learners put efforts into writing their work so that it would be read, understood and memorized. Additionally, SNS was used in a diverse set of locations and at varying times. The way the interviewees described these situations shows how integrating the tool into their daily lives enabled them to choose whether or not to work. The possibility for students to make these kinds of choices satisfied their need for autonomy [37]. This in turn may facilitate users’ engagement in learning tasks [38].

We postulate that these elements may explain the increased amount of time spent on personal work carried out with SNS, compared to what residents experience in traditional teaching sessions. The greater engagement when using SNS for teaching has already been raised in previous studies with students from multiple disciplines [39].

### A positive influence of remote interactions on face-to-face exchanges

Residents spent a significant amount of time checking SNS, but online discussions were quite limited. We postulate that autonomy in learning; personality differences and varying individual time constraints make it difficult or impossible for all learners to be fully involved in a learning situation at the same time.

On the other hand, when residents met, the group dynamics, and the sense of cohesion enabled all learners to engage in discussions. This may be appraised as a positive influence of SNS use in asynchronous distance learning on the quality of face-to-face exchanges. Previous work also identified the potential of SNS for knowledge transfer and collaborative working, by allowing students to understand questions, develop arguments, and share meaning and conclusions among themselves [30].

### Strengths and weaknesses

This study addresses the need to understand the particularities of SNS usage in family practice training. The theoretical framework, methodology and interview guide enabled us to collect relevant data in relation to our research objectives. Data saturation and investigator triangulation are important criteria in qualitative research.

Our study has several limitations. Although we believe that our findings can also be applicable to other fields of study, the scope and the methodology we used prevent any generalization and transferability of our results to other teaching and learning environments. Also, there are clear limitations to data saturation when purposive sampling is used, as saturation may occur sooner if all the interviewees belong to a similar subset. This could explain why we rapidly reached saturation. The investigator was the teacher of four of the eight participants, which is likely to influence their responses. Nevertheless, we did not observe any differences in the ways in which these participants expressed themselves during the interviews. As it doesn’t form part of the theoretical framework of social appropriation, we didn’t interview the participants specifically on their non-SNS and non-face-to-face collaborative activities as a means of capturing the third likely mechanism through which they could communicate/collaborate. This participants’ characteristic did not emerge from the interviews.

## Conclusions

The blended learning environment we studied may be an innovative alternate solution to the constraints relating to residents’ location and the lack of teachers in general practice. Our study allowed us to document a high level of social appropriation among SNS users. Furthermore, it seems that the way residents use SNS encourages face-to-face discussions, and that their high level of perceived autonomy fosters their engagement in learning tasks. Further studies are necessary to evaluate the effectiveness of SNS in blended learning environments and the appropriation of SNS by teachers.

## Additional file

Additional file 1: **Interview guide.** (DOCX 53 kb)

## References

[1] Starfield B (1994). Is primary care essential?. The Lancet.

[2] Chambe J, Maisonneuve H, Leruste S, Renoux C, Huas C (2014). Etat des lieux des procedures de validation du DES de médecine générale en France. Exercer.

[3] Hummers-Pradier E, Beyer M, Chevallier P, Eilat-Tsanani S, Lionis C, Peremans L (2009). The Research Agenda for General Practice/Family Medicine and Primary Health Care in Europe. Part 1. Background and methodology. Eur J Gen Pract.

[4] Allen J, Gay B, Crebolder H, Heyman J, Svab I, Ram P (2002). The European definitions of the key features of the discipline of general practice: the role of the GP and core competencies. Br J Gen Pract..

[5] Guldal D (2012). Educational expectations of GP trainers. A EURACT needs analysis. Eur J Gen Pract.

[6] Djalali S, Rosemann P (2012). Le cursus parfait de médecine générale - qui l’a donc inventé?. Prim Care.

[7] Gray K, Annabell L, Kennedy G (2010). Medical student’s use of Facebook to support learning: insights from four case studies. Med Teach.

[8] Garrison DR, Kanuka H (2004). Blended learning: Uncovering its transformative potential in higher education. Internet High Educ.

[9] Rowe M, Frantz J, Bozalek V (2009). The role of blended learning in the clinical education of healthcare students: A systematic review. Med Teach.

[10] Ellaway R, Masters K (2008). AMEE Guide 32: e-Learning in medical education Part 1: Learning, teaching and assessment. Med Teach.

[11] Seluakumaran K, Jusof FF, Ismail R, Husain R (2011). Integrating an open-source course management system (Moodle) into the teaching of a first-year medical physiology course: a case study. Adv Physiol Educ.

[12] Masters K, Ellaway R (2008). e-Learning in medical education Guide 32 Part 2: Technology, management and design. Med Teach.

[13] Conole G, De Laat M, Dillon T, Darby J (2008). ‘Disruptive technologies’, ‘pedagogical innovation’: What’s new - Findings from an in-depth study of students’ use and perception of technology. Comput & Educ.

[14] Gunawardena C, Hermans MB, Sanchez D, Richmond C, Bohley M, Tuttle R (2009). A theoretical framework for building online communities of practice with social networking tools. Educ Media Int..

[15] DeSchryver M, Mishra P, Koehleer M, Francis A (2009). Moodle vs. Facebook: Does using Facebook for Discussions in an Online Course Enhance Perceived Social Presence and Student Interaction?.

[16] Karpinski A, Duberstein A (2009). A Description of Facebook Use and Academic Performance Among Undergraduate and Graduate Students.

[17] Ross S, Lai K, Walton J, Kirwan P, White J (2013). “I have the right to a private life”: Medical students’ views about professionalism in a digital world. Med Teach.

[18] Cunningham A (2014). Social media and medical professionalism. Med Educ.

[19] Fenwick T (2014). Social media and medical professionalism: rethinking the debate and the way forward. Acad Med J Assoc Am Med Coll.

[20] Cartledge P, Miller M, Phillips B (2013). The use of social-networking sites in medical education. Med Teach.

[21] Cheston CC, Flickinger TE, Chisolm MS (2013). Social media use in medical education: a systematic review. Acad Med J Assoc Am Med Coll.

[22] Pander T, Pinilla S, Dimitriadis K, Fischer MR (2014). The use of Facebook in medical education-a literature review. GMS Z Für Med Ausbild.

[23] Proulx S, Vieira L, Pinede N (2005). Penser les usages des TIC aujourd’hui: enjeux, modèles, tendances. Enjeux et usages des TIC: aspects sociaux et culturels.

[24] Aubin I, Mercier A, Bauman L, Lehr-Drylewicz AM, Imbert P, Lettrilliart L (2008). Introduction à la recherche qualitative. Exercer..

[25] Barnett S, Jones SC, Bennet S, Iverson D, Bonney A (2012). General practice training and virtual communities of practice – a review of the literature. BMC Fam Pract.

[26] Barnett S, Jones SC, Caton T, Iverson D, Bennett S, Robinson L (2014). Implementing a Virtual Community of Practice for Family Physician Training: A Mixed-Methods Case Study. J Med Internet Res.

[27] Prensky M (2001). Digital natives, digital Immigrants. On the Horizon.

[28] Britten N (1995). Qualitative Research: Qualitative interviews in medical research. BMJ.

[29] Miles MB, Huberman AM (1994). Qualitative data analysis: An expanded sourcebook.

[30] Barnett S, Jones SC, Bennett S, Iverson D, Bonney A (2013). Perceptions of family physician trainees and trainers regarding the usefulness of a virtual community of practice. J Med Internet Res.

[31] Sircar F, Clauson KA, Duffy M, Joseph S (2012). Thematic analysis of pharmacy students’ perceptions of Web 2.0 tools and preferences for integration in educational delivery. Teach learn med.

[32] DeCamp M, Koenig TW, Chisolm MS (2013). Social media and physicians’ online identity crisis. JAMA.

[33] Hamid S, Waycott J, Kurnia S (2014). An empirical study of lecturers’ appropriation of social technologies for higher education. Australas J Educ Technol.

[34] Marquet P (2012). e-Learning et conflit instrumental. Rech & form.

[35] Richardson JC, Newby T (2006). The Role of Students’ Cognitive Engagement in Online Learning. Am J Distance Educ.

[36] Rotgans JI, Schmidt HG (2006). Cognitive engagement in the problem-based learning classroom. Adv Health Sci Educ Theory Pract.

[37] Deci EL, Vallerand RJ, Pelletier LG, Ryan RM (1991). Motivation and Education: The Self-Determination Perspective. Educ Psychol..

[38] Deci EL, Renninger KA, Hidi S, Krapp A (1992). The relation of interest to the motivation of behavior: A self-determination theory perspective. The role of interest in learning and development.

[39] Hamid S, Waycott J, Kurnia S, Chang S (2015). Understanding students’ perceptions of the benefits of online social networking use for teaching and learning. Internet High Educ.

