# Supplementary material for: How do general practice residents use social networking sites in asynchronous distance learning?
Source: BMC Med Educ. 2015 Sep 21;15:154. doi: 10.1186/s12909-015-0435-x (PMC4578679; doi:10.1186/s12909-015-0435-x)
Supplement: Additional file 1: — Interview guide. (DOCX 53 kb) [file 12909_2015_435_MOESM1_ESM.docx]

Interview guide

Age

Number of blended learning courses followed

Pedagogical aspects of the courses

Number of courses followed with Facebook

Integration of Facebook in the courses

Personal use of Facebook

Prior mastery of Facebook

Interaction with the tool, interaction with other people

Feeling of mastery of Facebook in the course

Moments of use for learning purposes (search for unexpected moments)

Place of use for learning purposes (search for unexpected places)

Spontaneity and ease of use for learning purposes

Contribution to the realization of the personal work

Difference between initial vision of Facebook for learning purposes and vision after the participation of this course
